# Experiences from treating seven adult 5q spinal muscular atrophy patients with Nusinersen

**DOI:** 10.1177/1756286420907803

**Published:** 2020-03-05

**Authors:** Elisabeth Jochmann, Robert Steinbach, Thomas Jochmann, Ha-Yeun Chung, Annekathrin Rödiger, Rotraud Neumann, Thomas E. Mayer, Klaus Kirchhof, Dana Loudovici-Krug, Ulrich C. Smolenski, Otto W. Witte, Julian Grosskreutz

**Affiliations:** Department of Neurology, Jena University Hospital, Am Klinikum 1, Jena, 07747, Germany; Department of Neurology, Jena University Hospital, Jena, Germany; Department of Computer Science and Automation, Technische Universität Ilmenau, Ilmenau, Germany; Department of Neurology, Jena University Hospital, Jena, Germany; Department of Neurology, Jena University Hospital, Jena, Germany; Department of Radiology, Section Neuroradiology, Jena University Hospital, Jena, Germany; Department of Radiology, Section Neuroradiology, Jena University Hospital, Jena, Germany; Department of Radiology, Section Neuroradiology, Jena University Hospital, Jena, Germany; Department of Physiotherapy, Jena University Hospital, Jena, Germany; Department of Physiotherapy, Jena University Hospital, Jena, Germany; Department of Neurology, Jena University Hospital, Jena, Germany; Department of Neurology, Jena University Hospital, Jena, Germany

**Keywords:** antisense oligonucleotide, intrathecal, Nusinersen, patient-reported outcome, spinal muscular atrophy

## Abstract

**Background:**

The antisense oligonucleotide Nusinersen recently became the first approved drug against spinal muscular atrophy (SMA). It was approved for all ages, albeit the clinical trials were conducted exclusively on children. Hence, clinical data on adults being treated with Nusinersen is scarce. In this case series, we report on drug application, organizational demands, and preliminary effects during the first 10 months of treatment with Nusinersen in seven adult patients.

**Methods:**

All patients received intrathecal injections with Nusinersen. In cases with severe spinal deformities, we performed computed tomography (CT)-guided applications. We conducted a total of 40 administrations of Nusinersen. We evaluated the patients with motor, pulmonary, and laboratory assessments, and tracked patient-reported outcome.

**Results:**

Intrathecal administration of Nusinersen was successful in most patients, even though access to the lumbar intrathecal space in adults with SMA is often challenging. No severe adverse events occurred. Six of the seven patients reported stabilization of motor function or reduction in symptom severity. The changes in the assessed scores did not reach a significant level within this short time period.

**Conclusions:**

Treating adult SMA patients with Nusinersen is feasible and most patients consider it beneficial. It demands a complex organizational and interdisciplinary effort. Due to the slowly decreasing motor functions in adult SMA patients, long observation phases for this recently approved treatment are needed to allow conclusions about effectiveness of Nusinersen in adults.

## Introduction

Spinal muscular atrophy (SMA) is a genetic disorder leading to degeneration of lower motor neurons, and, consequently, to severe and progressive muscle atrophy. SMA is not associated with cognitive impairment.^1^ The disease is classified into four phenotypes, defined by the age at onset and the highest attained developmental motor milestone (SMA I: never achieve unassisted sitting; SMA II: unassisted sitting; SMA III: unassisted walking; SMA IV: adult onset).^2^

**Figure 1.**
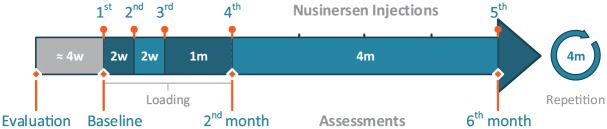
Treatment and assessment schedule. We began treatment shortly after a preparational day hospital evaluation. Within the first 2 months of treatment, four administrations of Nusinersen take place within the ‘loading phase’. The treatment has to be repeated every 4 months thereafter. Intrathecal injections of Nusinersen were conducted by neurologists and neuroradiologists, the assessments involved neurologists, physical therapists, the pulmonary unit, and the laboratory for blood and urine workup.

Until recently, treatment of patients with SMA was restricted to symptomatic approaches. In 2016, Nusinersen was approved as the first specific therapy for 5q-associated SMA in the United States, followed by the European Union in 2017. The effective molecule is an antisense oligonucleotide, which must be administered intrathecally every 4 months after loading (Figure 1). It has been approved for pediatric and adult patients, albeit the pivotal studies were conducted only with children.^3,4^ In the pivotal studies, children with severe scoliosis, or very limited motor function, were excluded. Adult SMA patients, however, often suffer from both of these conditions, which leads to complex challenges in clinical approach.

Data of adults being treated with Nusinersen is scarce.^5^ Thus far, Stolte and colleagues and Wurster and colleagues described the feasibility and safety of lumbar puncture for the application of Nusinersen in adult patients with SMA.^6,7^ Walter and colleagues recently reported the treatment effects in a first adult SMA type III cohort.^8^ Apart from that cohort, no follow-up data describing the clinical course of adult patients receiving treatment with Nusinersen has been published to date, and no reports on adult SMA type II patients exist so far. Here, we report on the first 10 months of treatment of seven adults with SMA type II and III, with focus on drug application, organizational demands, patient characteristics, and preliminary effects.

## Methods

Seven patients aged 20–68 years were treated with Nusinersen for at least 10 months. We initially evaluated the patients in the neurologic day clinic including thorough patient information and treatment planning. Treatment planning comprised, for example, a spine CT, pulmonary function assessment, and a request of full cost coverage by the health insurance company to avoid later cancellation of hospital costs. Subsequently, the patients were admitted to the Department of Neurology at Jena University Hospital for each application.

Treatment of our adult patients was conducted by a multidisciplinary team, comparable to the description by Sansone and colleagues for the treatment of children.^9^ Our team includes neurologists (preparational evaluation, definition of individual therapeutic goals, organization and documentation of the treatment, and intrathecal application of Nusinersen in patients without major spinal deformities), neuroradiologists (fluoroscopic and CT-guided intrathecal application), and physical therapists (physiotherapeutic assessments).

All participants provided written informed consent for publication of the data in an international medical journal. Confirmation was obtained from the ethics committee of Jena University Hospital, Jena, Germany, that this case series does not require ethical approval.

### Drug administration

We applied Nusinersen intrathecally lumbar, following the prescribing information. In three patients with SMA type III who were able to sit unassisted or with assistance of one person, lumbar puncture was conducted on the ward in a sitting position without local anesthetic or sedation. In the remaining four patients, CT-guided lumbar punctures were performed in a lateral position by experienced neuroradiologists with assistance by neurologists with constant cardiopulmonary monitoring. Three of these four patients were injected by transforaminal access.^10^ In one patient, we switched from fluoroscopic guidance to CT-guided transforaminal applications due to lack of cerebrospinal fluid backflow at the fourth injection. Severely affected patients were brought in position with the help of personal assistants or family members in order to make them as comfortable as possible. If requested by the patient, a local anesthetic (Lidocaine) was applied.

In two additional patients for whom Nusinersen application was planned in the reported time period, treatment was cancelled after an unsuccessful first procedure. In one of the two, CT-guided lumbar puncture failed due to metal implants and calcification, in the other, the intervention was discontinued due to anxiety and pain.

### Assessments

We evaluated all patients before, and at 2, 6, and 10 months after the beginning of the treatment (Table 1, Figure 1). Motor function was assessed with the Hammersmith Functional Motor Scale Expanded (HFMSE),^11^ the Revised Upper Limb Module (RULM),^12^ and the 6-Minute Walk Test (6MWT).^13^ When scoring the RULM and the HFMSE, evaluators rated if the motor tests were limited by contractures. Within the basic assessment of the RULM, they additionally documented the existence of elbow contractures. Evaluators were either trained directly for RULM, HFMSE, and 6MWT in a dedicated workshop, instructed by trained evaluators, or studied the instruction manuals. Physical functioning in activities of daily living was evaluated by the Revised Amyotrophic Lateral Sclerosis Functional Rating Scale (ALSFRS-R).^14^ Furthermore, pulmonary function was measured and extensive laboratory blood and urine workup was carried out. To achieve comparability, and to contribute to a multicenter registry, our clinical evaluations complied with national recommendations. In addition, quality of life was documented using EUROQoL EQ-5D; Patient-Reported-Outcome was scored with the Measure Yourself Medical Outcome Profile 2 (MYMOP-D, German translation of MYMOP2).^15^

**Table 1.** Patient characteristics and important assessments. The seven adult SMA patients exhibit high demographic and clinical heterogeneity. Patient 3 was the only patient who could walk unassisted; thus, he was the only patient who could perform the 6MWT. As the 10-month 6MWT could not be evaluated due to an error in documentation, data from the 14-month assessment are presented. Patient 4 received only four applications of Nusinersen due to a sacral pressure ulcer, and subsequent withdrawal from treatment. As patient 4 is bedridden, no spirometry could be performed in our pulmonary unit. Six patients reported subjective improvements in symptom severity that partly coincided with the assessed scores. Clinically meaningful improvements in the assessments are indicated, with the absolute value of increased points between baseline and last assessment (as RULM and HFMSE scores are on an ordinal scale, no percentage is calculated); improvement in the 6MWT is indicated as a percentage.

	Patient 1	Patient 2	Patient 3	Patient 4	Patient 5		Patient 6		Patient 7	
Demographics										
Sex	Male	Female	Male	Male	Male		Female		Male	
Age at baseline	45 years	50 years	20 years	57 years	68 years		31 years		22 years	
Clinical characteristics										
SMA type	III	II	III	II	III		II		II	
SMN2 copy number	Not known	2	>4	Not known	4		3		2	
Type of mutation in SMN1 as stated in genetic report	Homozygote deletion exon 7, heterozygote deletion exon 8	Homozygote deletion exons 7 and 8	Homozygote deletion exons 7 and 8	Homozygote deletion exons 7 and 8	Homozygote deletion exon 7, heterozygote deletion exon 8		Homozygote deletion (no further details stated)		Deletion exons 7 and 8 (no further details stated)	
Age at onset	7–8 years	9 months	14 years	1–1.5 years	14 years		9 months		1 year	
Best motor milestone in patient history	Unassisted walking	Assisted walking	Unassisted walking	Assisted walking	Unassisted walking		Unassisted sitting		Assisted standing	
Mobility and dependence at baseline	Wheelchair-bound, unassisted transfers	Wheelchair-bound, 24 h assistance	Ambulatory	Mostly bedridden, 24 h assistance	Wheelchair-bound, unassisted transfers		Wheelchair-bound, 24 h assistance		Wheelchair-bound, 24 h assistance	
Need for ventilatory support	No	Indication at night since age 49, not frequently used	No	8–10 h a day, since age 53	At night since age 50 (concurrent obstructive sleep apnea)		Indication at night since age 31, not frequently used		No	
Gastrostomy	No	No	No	After 4th application	No		No		No	
Scoliosis	Yes	Yes	No	Severe	No		Yes, posterior spinal fusion since childhood		Yes, posterior spinal fusion since childhood	
Drug administration										
	Lumbar puncture on ward	Fluoroscopic guidance, since 4th application CT-guided, transforaminal	Lumbar puncture on ward	CT-guided, interlaminar	Lumbar puncture on ward		CT-guided, transforaminal		CT-guided, transforaminal	
Assessments during treatment with Nusinersen (excerpt)										
RULM (total score of 37 points), assessed by physical therapist										
Baseline	15	4	37	1	**17**		**5**		**3**	
At 2nd month	16	7	37	0	**35**		**18**		**12**	
At 6th month	15	5	35	n/a	**35**		**10**		**12**	
At 10th month	15	5	35	n/a	**37**	⇧ +20/37	**16**	⇧ +11/37	**19**	⇧ +16/37
Elbow contracture	No	Yes	No	Yes	No		Yes		Yes	
Limitation by contracture	No	No	No	No	No		No		No	
HFMSE (total score of 66 points), assessed by physical therapist										
Baseline	29	0	60	0	**6**		**2**		**0**	
At 2nd month	28	0	60	0	**10**		**2**		**3**	
At 6th month	28	0	56	n/a	**11**		**0**		**3**	
At 10th month	28	0	63	n/a	**23**	⇧ +17/66	**7**	⇧ +5/66	**6**	⇧ +6/66
Limitation by contracture	No	No	No	No	No		No		No	
6MWT (meters walked), assessed by physical therapist										
Baseline			**275** m							
At 2nd month			**305** m							
At 6th month			**327** m							
At 10th month			n/a							
At 14th month			**343** m ⇧ +25%							
ALSFRS-R (total score of 48 points), subscores: (bulbar/upper limb/lower limb/respiratory), assessed by neurologist										
Baseline	36 (12/ 8/ 4/ 12)	19 (10/ 1/ 0/ 8)	45 (11/ 12/ 10/ 12)	15 (9/ 0/ 0/ 6)	24 (8/ 4/ 2/ 10)		30 (12/ 6/ 0/ 12)		27 (12/ 3/ 0/ 12)	
At 2nd month	35 (11/ 9/ 5/ 10)	20 (10/ 2/ 0/ 8)	44 (11/ 12/ 9/ 12)	15 (9/ 0/ 0/ 6)	26 (8/ 6/ 2/ 10)		30 (11/ 7/ 0/ 12)		27 (12/ 3/ 0/ 12)	
At 6th month	33 (11/ 7/ 3/ 12)	21 (10/ 3/ 0/ 8)	44 (11/ 12/ 9/ 12)	n/a	28 (11/ 5/ 2/ 10)		32 (12/ 8/ 0/ 12)		29 (12/ 3/ 2/ 12)	
At 10th month	35 (11/ 8/ 4/ 12)	21 (10/ 3/ 0/ 8)	44 (11/ 12/ 9/ 12)	n/a	27 (9/ 5/ 3/ 10)		n/a		29 (12/ 3/ 2/ 12)	
Spirometry: FVC (% of predicted), assessed by pulmonary unit										
Baseline	n/a	44	111	n/a	101		31		15	
At 2nd month	86	n/a	n/a	n/a	n/a		30		27	
At 6th month	79	45	115	n/a	105		31		25	
At 10th month	81	43	110	n/a	93		32		24	
EUROQoL EQ-5D-5L Index, self-evaluated by patient										
Baseline	0.39	–0.02	0.60	0.06	0.06		0.18		0.18	
At 2nd month	0.49	0.06	0.81	0.18	0.15		0.18		0.18	
At 6th month	0.43	0.09	0.81	n/a	0.13		0.06		0.18	
At 10th month	0.77	0.09	0.81	n/a	0.13		n/a		0.18	
Subjective changes of symptoms and motor function after 10 months of treatment with Nusinersen, as reported by patient										
	⇧	⇧	⇧		⇧		⇧		⇧	
At 10th month	Strength of arms ↑; facilitation of transfers, sitting, unsupported standing, regain of walking with walking frame up to 10 m	Strength of hands, forearms, and chewing muscles ↑; neck stability ↑; less dysphagia; louder voice, clearer speech	Total walking distance ↑; less fatigability	After 2 months: Strength of right hand ↓ (last remaining motor function in extremities); less dysphagia	Strength of shoulders and arms ↑; facilitation of transfers and rolling over; regain of unassisted standing		Strength of arms, hands, right knee extension and flexion ↑; less assistance needed for eating		Strength of right hand ↑; neck stability ↑; stronger cough; regain of unsupported sitting; less dysphagia, louder voice; less fatigue	
Further assessments	Validated German version of the MYMOP-D, peak cough flow (both self-assessed, weekly)									

⇧ marks a tendency of improvement over the 10 months of treatment with Nusinersen, ↑ and ↓ mark increase/improvement and decrease/deterioration, respectively.

ALSFRS-R, Revised Amyotrophic Lateral Sclerosis Functional Rating Scale; CT, computed tomography; FVC, forced vital capacity; HFMSE, Hammersmith Functional Motor Scale Expanded; MYMOP-D, Measure Yourself Medical Outcome Profile; 6MWT, 6-minute Walk Test; RULM, Revised Upper Limb Module; SMA, spinal muscular atrophy; SMN, spinal motor neural protein.

## Results

Between August 2017 and May 2019, seven adult patients (42 ± 18 years) with SMA type II (*n* = 4) and III (*n* = 3) were treated with Nusinersen in our hospital. For the reported treatment period of 10 months, six of the patients received the entire six doses of Nusinersen. These six patients reported regaining of motor functions, appreciable in their daily life (for details see Table 1). Patient 4 was not able to receive the fifth and consecutive applications of Nusinersen due to the development of a sacral pressure ulcer close to the injection site. This patient reported decreasing strength but less dysphagia at 6 months and decided to discontinue the treatment with Nusinersen. In a routine follow up at 12 months after initiation of Nusinersen, we conducted the *ALSFRS-R*, which decreased by 1 point due to the percutaneous endoscopic gastrostomy he had received meanwhile.

### Drug administration

Intrathecal administration of Nusinersen was feasible in seven out of nine patients in the reported time period (78%).

A total of 18 conventional interlaminar lumbar punctures were performed on the ward. Overall, lumbar punctures in these patients did not require more attempts than in healthy individuals.

A total of 19 CT-guided applications were performed, 15 with a transforaminal approach. Cardiopulmonary monitoring documented stable heart activity and oxygen saturation throughout the procedures with no need for respiratory support.

Three lumbar punctures with fluoroscopic guidance were successful. Fluoroscopic guidance was arduous in presence of severe spinal deformities, postoperative alterations, and demineralization.

### Adverse events

No severe adverse events were observed after 40 applications of Nusinersen. The rate and nature of adverse events (headache after lumbar puncture in two patients, proteinuria in one patient) are in accordance with the literature.^6,7,10^

### Assessments

The ALSFRS-R, pulmonary assessments, EQ-5D index, and laboratory assessments showed no clinically meaningful changes for any of the seven patients.

**Figure 2.**
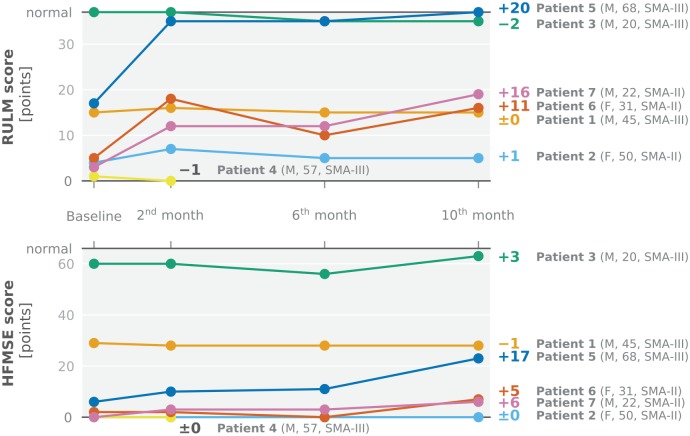
Changes in RULM and HFMSE scores during the first 10 months of treatment with Nusinersen. Absolute values of the (top) RULM score (total range 0–37 points, higher indicates better) and the (bottom) HFMSE score (total range 0–66 points, higher indicates better) as well as their changes between baseline and 10th month are depicted for each individual patient. Patients are briefly characterized in brackets: sex, age in years at baseline, type of SMA. RULM (top) and HFMSE (bottom) scores showed clinically meaningful changes in three patients (patient 5, 6, 7), with RULM score depicting an increase before HFMSE score (2 versus 10 months after initiation of the treatment). Patient 3, who is ambulatory, already started with a RULM score of 37 points (maximum score) at baseline. Patient 4 discontinued treatment before the fifth application (and herewith 6th month assessment) of Nusinersen due to the development of a sacral pressure ulcer. HFMSE, Hammersmith Functional Motor Scale Expanded; F, female; M, male; RULM, Revised Upper Limb Module; SMA, spinal muscular atrophy.

In contrast, the RULM, designed to score motor function of the upper limb (range 0–37), depicted clinically meaningful improvements in three patients as soon as 2 months after starting the treatment (Figure 2, top). Their scores increased from baseline to 10 months of treatment by an average of +15.7 points (SD 4.5); the mean change for all six patients receiving six doses of Nusinersen was +7.7 (SD 9.3). The HFMSE, used for the assessment of overall physical abilities in SMA type II and III, showed an increase in the same three patients after 10 months, with an average of +9.3 points (SD 6.7) (Figure 2, bottom). The mean change for all six patients was +5 (SD 6.5). The only patient able to perform the 6MWT walked 275 m at baseline, 327 m at 6 months, and 343 m at 14 months (+25%). MYMOP-D was performed by three patients (2, 5, and 7) throughout the first 10 months of treatment for weekly self-evaluation. In patient 2, we documented increasing scores by more than 1 point in two out of four items referring to improvement of handwriting and general wellbeing after 6 months.

All four patients with SMA type II had elbow contractures. The three patients with SMA type III had no documented contractures. The evaluators considered none of the motor function tests performed by any of the patients as limited by contractures beyond the limitations due to the pareses.

Three out of four SMA type II patients described dysphagia at baseline. These three patients reported a subjective decrease of dysphagia within the first 10 months of application of Nusinersen.

Patient 1, who showed stable results in the assessments during the first 10 months of treatment (Figure 2, Table 1), meanwhile received Nusinersen for a total of 22 months (9 doses). At his 14-month assessment, the RULM score increased by 11 points as compared with baseline, and stayed at this level until now. HFMSE score increased at his 22-month assessment (8 months later than the RULM score) by 9 points as compared with baseline.

## Discussion

Defining individual treatment goals prior to initiating treatment with Nusinersen was of major importance in our center. Due to the lack of data regarding the effectiveness of Nusinersen in adult patients, the decision to continue treatment after 1 year will take the realization of these goals into account.

Most of our patients defined stabilization of their current clinical state as the major therapeutic expectation, which is consistent with observations elsewhere.^16^ They emphasized breathing and hand motor function.

We performed multiple assessments to monitor the clinical condition and quality of life of the patients undergoing treatment. Due to the small and heterogenous cohort of patients reported in this case series, and the short time period of documentation, no statistically substantiated statements about clinical benefits can be drawn from the assessments. It is important to note that, before treatment, the patients had a progression of disease for, on average, 36 years since onset of symptoms. All patients reported that, over the course of the 3 years preceding the start of treatment with Nusinersen, their symptoms and conditions had become worse.

Six out of seven patients described subjective improvements in motor skills since the treatment with Nusinersen, which were also acknowledged by personal assistants and treating physical therapists (Table 1). In the six patients with improvements, only three had increasing RULM and HFMSE scores, with HFMSE increasing later and to a lesser degree (Figure 2). One other patient’s RULM (patient 3, SMA type III) was already at the maximum possible score at the start, and thus could not improve. His HFMSE score was close to the maximum possible score, and fluctuated around a high level from test to test with no distinct tendency to increase or decrease. Comparable courses of HFMSE in SMA type III patients with large initial HFMSE scores, and improvements in the 6MWT, have been described by Darras and colleagues.^17^ It could be assumed that the steps to improve HFMSE are higher with large scores.^17^ At that level, skills like squatting or jumping are tested, which might take a longer time of training even if motor function had improved in the meantime.

For the remaining two patients, the lack of increasing RULM (and HFMSE) scores despite subjective motor improvements could be explained by the circumstance that, over time, they have developed strategies to optimally employ their remaining motor functions. This leads to appreciable improvements in function even with marginal increase in strength. The coarse gradings in the assessed motor scores, however, do not reflect these nuances.

Interestingly, in the three patients with increasing scores, the RULM score had already increased at the first assessment, 2 months after treatment initiation, while the HFMSE did not depict similar results before the 10 month assessment. We see three possible explanations for this finding: first, it could be interpreted as RULM being more sensitive to subtle changes in motor functions than HFMSE. Second, since the three patients with increasing scores we report on had more remaining motor function in the upper than in the lower limbs, it could be explained by an easier and earlier recovery of motor function in areas with more muscle strength left. Third, and more speculatively, changes in motor function in adults under treatment with Nusinersen could generally initially occur in the upper limbs due to the shorter distance between motor neurons and muscles compared with the lower limbs. Thus, RULM, which was designed to document upper limb motor function, can depict increasing scores early on. HFMSE, with motor tests aimed at overall motor activities, including many items involving the lower limbs (with a longer distance between motor neurons and muscles), could therefore document changes only after a longer time period.

In the patients with notable increase in the RULM and HFMSE scores, no clear pattern of patient characteristics can be deduced. They were of different age (22, 31, 68 years old), had different types of SMA (II and III), and different SMA2 gene copy numbers (2, 3, and 4). In contrast to our observations, a correlation between duration of disease and response to treatment, as well as a correlation between age and response to treatment, was reported in children.^4^ On the other hand, Walter and colleagues also observed a lack of correlation in their adult cohort.^8^ The adult cohorts described so far are too small to generalize; however, there is no indication thus far that older age or longer disease duration in adults impacts the treatment response negatively.

RULM and HFMSE scores increased in three of the patients of our cohort within the first 10 months of treatment. Some of the assessed scores increased remarkably. Individual patients with outstanding improvements in motor assessments have also been seen by others in children and adults.^4,8^ The mean changes in RULM and HFMSE scores of our small heterogenous cohort lie within the same dimensions as data reported elsewhere. That is, the least-squares mean increase in HFMSE score after 15 months described in children with later-onset SMA (3.9 points) is comparable with the mean increase of all patients in our cohort after 10 months (5 points).^4^ Of children in the Nusinersen group in the latter study, 57% had an increase in HFMSE score after 15 months, which is also consistent with our data of adult patients after 10 months. Nevertheless, the observed individual increases in motor scores remain astonishingly high, as increases in the dimension described in children were not expected, even in individual patients. Possible explanations for the observed improvements in motor scores could be placebo effect, the learning curve for the motor tests, and an increased frequency and intensity of physiotherapy due to increased motivation after starting a novel therapy. Furthermore, fluctuations in motivation and general condition could lead to altered results. On a biological level, this may indicate a nonfunctional recoverable state of motor neurons, which warrants further in-depth analysis of single motor units. Overall, the data suggest different individual responses to treatment in adults, similar to what has been described in children.

SMA type II patients all develop lower extremity contractures to some degree, which can have negative impact on the performance in HFMSE (SMA type III patients generally develop lower extremity contractures only to a minimal degree).^18^ This observation in SMA type II patients may also be transferred to the upper extremity and RULM. By leading to an impaired range of motion, contractures could be detrimental for motor testing in RULM. Several items of the RULM require the ability to fully extend the elbows to achieve the highest score. This could limit the achievable scores in patients at higher strength levels with elbow contractures. In our cohort, all SMA type II patients had documented elbow contractures. Nevertheless, as our assessors considered none of the motor function tests performed by any of the patients to be additionally limited by contractures, there is no indication that their treatment benefit was diminished by contractures within our observational period.

An improvement in the 6MWT in ambulatory SMA patients receiving Nusinersen has been described before. In a cohort of later-onset SMA children, Darras and colleagues reported an average of 30 m response per year, with an approximately linear increase over the course of about 3 years of treatment.^17^ Furthermore, Walter and colleagues described 64% of 11 adult patients improving their walking distance in the 6MWT by 31 m or more after 10 months of treatment.^8^ The increased walking distance in the ambulatory patient of our cohort (68 m after 14 months) is consistent with the described findings.

In general, motor assessments that are used for the evaluation of treatment effects of Nusinersen should be sensitive enough to detect small changes in motor function. In contrast to the RULM, the HFMSE, and the 6MWT, the ALSFRS-R score (which is not validated for SMA) remained stable for all patients within our observational period. Our data is consistent with another report of the first 10 months of Nusinersen treatment in adults.^8^ This points towards a rather minor potential of the ALSFRS-R to reflect a short-term therapeutic improvement in SMA patients.

A decrease or stability in dysphagia and thereby a decrease or stability in the risk for aspirational pneumonias and the need for nutritional support (percutaneous endoscopic gastrostomy) is a major therapeutic aim for most of the weaker SMA type II patients.^16^ The three patients from our cohort with dysphagia at baseline reported a subjective decrease in dysphagia within the first 10 months of treatment with Nusinersen. To provide objective results considering dysphagia, repeated swallowing studies would be needed in future assessments. With Nusinersen being approved for adults, no data derived from a placebo-controlled trial will be available. In this situation, clinical observations, as presented in this case series, are the best possible data with which to evaluate effectiveness. Thus, an inevitable limitation of the data presented is that a placebo effect leading to increasing scores cannot be excluded as the data was deduced from clinical data from an approved treatment and not from a controlled clinical trial.

## Implications for clinical care

Treatment of adult SMA patients with Nusinersen is feasible. Most patients consider it beneficial and seek to continue the treatment, despite the burden of undergoing the unpleasant and inconvenient procedure. It imposes a considerable organizational effort, including the setup of a well-structured treatment plan in close coordination with the patients as well as the establishment of a multidisciplinary medical team.

To date, no predictors of effectiveness of treatment with Nusinersen in adults exist. In our center, we offer treatment to all 5q SMA patients, irrespective of age, type of SMA, SMA2 gene copy number, or clinical status for 1 year initially, after which evaluation of effectiveness takes place, taking into account the individual predefined treatment goals, motor assessments, and patient-reported outcome. Adult SMA patients with a long medical history, and often with motor conditions decreasing slowly over years, will need long observation phases in order to allow conclusions about effectiveness of treatment. This is underlined by the data presented for patient 1, whose RULM and HFMSE scores did not increase until well over a year after treatment initiation, while reporting subjective improvements beforehand.

Time, and increasing numbers of treated adults after the, only recent, approval of this treatment, will show whether the currently established motor assessments are able to sufficiently depict clinical status in this group of patients, and if Nusinersen can stabilize, or even improve, the situation for adult patients to a similar degree as is the case for children.
